# A Novel 24 h × 7 Days Broken Wire Detection and Segmentation Framework Based on Dynamic Multi-Window Attention and Meta-Transfer Learning

**DOI:** 10.3390/s25123718

**Published:** 2025-06-13

**Authors:** Han Wu, Shiyu Xiong, Yunhan Lin

**Affiliations:** 1 School of Computer Science and Technology, Wuhan University of Science and Technology, Wuhan 430081, China; hanwu@wust.edu.cn (H.W.); xiongshiyu@wust.edu.cn (S.X.); 2 Hubei Province Key Laboratory of Intelligent Information Processing and Real-Time Industrial System, Wuhan University of Science and Technology, Wuhan 430081, China; 3 Institute of Robotics and Intelligent Systems, Wuhan University of Science and Technology, Wuhan 430081, China

**Keywords:** Yolov11, segmentation, detection, attention mechanism, transfer learning

## Abstract

Detecting and segmenting damaged wires in substations is challenging due to varying lighting conditions and limited annotated data, which degrade model accuracy and robustness. In this paper, a novel 24 h × 7 days broken wire detection and segmentation framework based on dynamic multi-window attention and meta-transfer learning is proposed, comprising a low-light image enhancement module, an improved detection and segmentation network with dynamic multi-scale window attention (DMWA) based on YOLOv11n, and a multi-stage meta-transfer learning strategy to support small-sample training while mitigating negative transfer. An RGB dataset of 3760 images is constructed, and performance is evaluated under six lighting conditions ranging from 10 to 200,000 lux. Experimental results demonstrate that the proposed framework markedly improves detection and segmentation performance, as well as robustness across varying lighting conditions.

## 1. Introduction

Defect detection is a common issue in industrial scenarios [1]. In substations, securely attaching a grounding rod to high-voltage wires for safety and stability testing requires the detection of damaged conductors. Subsequently, segmentation masks of the detected damaged conductors must be extracted to enable precise pose estimation, which is critical for substation maintenance operations. However, traditional manual inspection methods fall short in terms of efficiency, adaptability to complex environments, and limitations within constrained operational spaces, making them inadequate for the demands of modern power system maintenance. Although infrared imaging and optical detection technologies have seen gradual advancements, their performance remains limited under adverse weather conditions and in the detection of subtle damages. Infrared inspection robots rely primarily on thermal information [2], while automated detection systems exhibit limited adaptability in complex scenarios [3].

With the continuous advancement of deep learning and computer vision technologies, visual models have achieved remarkable progress in object detection and segmentation tasks. However, in industrial scenarios such as substations, existing methods still face numerous challenges in practical deployment. On one hand, the sparsity of 3D point clouds in long-range perception limits detection accuracy. On the other hand, 2D visual models exhibit poor robustness under complex lighting conditions—particularly at night or in environments with drastic illumination changes—resulting in a significant decline in detection performance and limiting their capacity for reliable all-weather operation [4]. Furthermore, performance is constrained by the scarcity of high-quality annotated data [5,6], as well as the interference caused by complex backgrounds and lighting variations, which hinders effective visual feature extraction [7].

To enhance model generalization in complex environments, recent studies have combined image enhancement techniques with optimizations to the YOLOv8 model and introduced meta-learning strategies to address challenges related to few-shot learning and task heterogeneity [8]. By measuring task similarity and incorporating prior knowledge transfer, meta-learning has significantly improved model robustness under varying illumination and weather conditions, while also reducing dependence on large-scale annotated datasets and enhancing adaptability to novel classes. In addition, the application of image enhancement in low-contrast images has been shown to improve the effectiveness of feature extraction [9].

To address the challenges posed by various complex scenarios in substation maintenance—such as low model accuracy due to the near fusion of targets and background under low-light conditions at night, poor generalization under variable illumination, and the difficulty of acquiring comprehensive datasets in industrial environments—this paper proposes a novel broken wire framework tailored for substation applications. The proposed framework consists of an image enhancement module that mitigates the loss of contrast and blending between wires and background in low-light conditions, thereby improving segmentation and detection accuracy; an image detection and segmentation module that enhances generalization and robustness under varying illumination; and a meta-transfer learning framework designed to address the limitations of small-sample training and the negative transfer effect often observed in traditional transfer learning. The experiments, conducted under a wide range of lighting conditions throughout the day, validate the effectiveness of each module.

Experimental results demonstrate that the proposed framework expands the operational applicability in a substation, alleviates issues related to limited datasets and negative transfer in traditional learning schemes, and significantly improves detection and segmentation performance in dynamic lighting environments. The primary contributions of this work are summarized as follows:

- A novel framework for broken wire detection and segmentation in substation maintenance is proposed, incorporating an image enhancement module, an image detection and segmentation module, and a transfer learning-based training module. This framework extends broken wire inspection capabilities beyond standard light conditions to include low-light nighttime and high-exposure midday environments, offering a reliable 24 × 7 operational solution.
- To address challenges posed by complex backgrounds and insufficient sample sizes, an improved YOLOv11n is introduced, enhanced with a DMWA (Dynamic Multi-scale Window Attention). This significantly boosts detection and segmentation accuracy in challenging backgrounds. Additionally, a multi-stage meta-transfer learning is proposed, enabling rapid convergence on small-sample datasets while mitigating the negative transfer effects of conventional transfer learning. Under variable illumination conditions, the proposed visual module achieves a 41–73% improvement in performance compared with YOLOv11n.
- Experiments with various algorithms across the image enhancement, detection, and segmentation, and transfer learning modules demonstrate the effectiveness of the proposed framework. Ablation studies verify that performance improvements under low-light conditions are largely driven by the image enhancement module. The framework significantly enhances segmentation accuracy while ensuring stable inference across diverse architectures, thereby extending the operational range and duration of substation maintenance.

## 2. Related Work

In this section, we briefly review the core techniques integrated into the proposed framework, including wire segmentation and detection, sliding window attention mechanism, transfer learning, and image enhancement. These components are jointly designed to address challenges posed by low-light conditions, complex backgrounds, and limited annotated data in substation maintenance scenarios.

### 2.1. Wire Detection and Segmentation

In recent years, significant progress has been made in the detection and segmentation of damaged power wires in both 2D and 3D domains. However, real-world applications still face persistent challenges due to complex backgrounds, subtle damage patterns, limited training samples, and highly variable lighting conditions, which often result in loss of RGB information, low contrast, and strong reflections. The primary difficulty in identifying and segmenting damaged power lines lies in effectively learning robust damage features that can resist interference caused by variations in viewing angles, occlusion, and illumination.

In the context of 2D detection and segmentation, Hota et al. [5] improved wire detection accuracy using a CNN trained on multispectral images. Abdelfattah et al. [6] introduced generative adversarial networks for power-line segmentation, which enhanced the segmentation performance of small targets; however, both approaches suffered from limited generalization due to small-scale datasets. Yang et al. [7] proposed an attention fusion module to boost segmentation performance, though its effectiveness under complex backgrounds remains limited. Xu et al. [10] developed LinE segment TRansformers, which employ a multi-scale encoder–decoder architecture to enhance detection capabilities, albeit at the cost of increased computational demands. Damodaran et al. [11] leveraged Canny edge detection and Hough transforms to improve both accuracy and robustness, but further optimization is still needed.

For 3D power-line detection and segmentation, Nardinocchi et al. [12] used geometric assumptions for point cloud analysis, but sparse wire point clouds often led to erroneous detections. Yermo et al. [13] applied elevation filtering and 3D Hough transforms with non-maximum suppression on point clouds, though the computational overhead of Hough-based methods limits their scalability. Qin et al. [14] employed CIR-based LiDAR patterns to improve extraction accuracy, but their robustness in real-world environments remains unverified. Huang et al. [15] introduced a densely connected network combining local and global feature enhancement with elevation attention and Transformer architectures to fuse local and global information, significantly improving point cloud segmentation and multi-scale feature extraction, though the Transformer’s high computational cost is a drawback.

In hybrid 2D–3D approaches, Stambler et al. [16] proposed Deep Wire CNN, which performs 2D wire detection, followed by 3D reconstruction using aerial measurement data. However, limited fields of view and dataset constraints hinder its generalization. Kolbeinsson et al. [4] developed a monocular end-to-end model combining 2D segmentation and 3D depth estimation, achieving promising performance on synthetic datasets, yet lacking robustness under extreme lighting and complex environments. Muñoz et al. [17] utilized a stereo vision system combining UAV-captured 2D imagery and 3D point clouds to significantly improve wire detection accuracy and obstacle avoidance capabilities, though additional obstacle and hazard information is required to optimize system performance.

While 2D detection and segmentation are highly sensitive to spectral resolution, RGB information quality, and background complexity, 3D approaches often struggle with sparse point clouds and unstable performance in complex environments, making it difficult to identify subtle damage. Hybrid 2D–3D methods show promise by leveraging segmentation masks from RGB images and relatively environment-invariant depth maps to achieve more accurate 3D matching, thereby offering greater robustness, accuracy, and application potential. Nonetheless, challenges such as complex backgrounds, drastic illumination changes, and the scarcity of damaged-wire samples continue to limit detection and segmentation performance and robustness.

### 2.2. Sliding Window Attention Mechanism

The sliding window attention mechanism has shown strong capability in feature extraction and local information enhancement across tasks such as computer vision and time series analysis. SWA-Net [18] applies a feature-level sliding window strategy to prevent information loss from fixed-size patches, and introduces Local Feature Enhancement and Adaptive Feature Selection modules to enrich fine-grained features and dynamically emphasize key regions, improving FER performance in complex environments. Neighborhood Attention (NA) further reduces attention complexity from quadratic to linear while preserving spatial structure via shift equivariance [19]. Based on NA, the NAT Transformer expands the receptive field and lowers computational cost in image classification and object detection tasks.

ASHFormer combines high-resolution networks with a sliding window self-attention block to enhance both long-range and local feature interactions in well-log stratigraphic correlation, leading to improved matching accuracy [20]. In time series applications, the sliding window-based two-stage decomposition method [21] leverages a two-stage decomposition based on sliding windows to extract key fluctuation features, thereby enhancing short-term wind speed prediction. The Swin Transformer integrates sliding window attention to overcome the limited global modeling capacity of traditional CNNs in gaze estimation tasks [22]. In addition, sliding window attention and high-response feature reuse dynamically adjust the perceptual scope of features using sliding window attention and incorporate a high-response feature reuse mechanism, significantly improving multimodal emotion recognition through more effective information fusion [23].

Overall, sliding window attention preserves local feature integrity under multi-scale conditions while balancing computational efficiency and model performance, offering robust feature representation and recognition capabilities for both visual and time-series tasks.

### 2.3. Transfer Learning

Transfer learning aims to facilitate the learning of a target task by leveraging knowledge acquired from a source domain, including both data distributions and task-related features [24]. TrAdaBoost [25] achieves knowledge transfer by employing a sample reweighting strategy, which utilizes a small amount of labeled data from the target domain to optimize a maximum entropy classifier, thereby improving classification performance. Transfer learning has also demonstrated significant advantages in image classification and object detection tasks, enhancing model adaptability [26] and boosting small object detection capabilities through cross-dataset knowledge transfer [27].

In industrial scenarios, transfer learning is widely applied to feature alignment and cross-domain adaptation. Deep convolutional adaptation methods [28], which incorporate Maximum Mean Discrepancy, reduce discrepancies in damage feature distributions, thus improving detection generalization. A CNN-Transformer hybrid model [29] employs grayscale feature map transfer learning to enhance cross-domain performance in steel wire rope defect diagnosis.

However, conventional transfer learning may lead to negative transfer, where transferred knowledge hinders rather than helps the target task. Meta-transfer learning has been proposed to mitigate this issue, especially under few-shot learning conditions. A task-aware meta-learning framework [30] improves task selection through feature clustering, thereby enhancing low-sample adaptability in structural damage segmentation. Attention-based deep meta-transfer learning [31] introduces a parameter modulation strategy based on meta-learning for fine-grained fault diagnosis, significantly improving recognition across different devices. Nevertheless, the training process of meta-transfer learning typically demands considerable computational resources, and further work is needed to optimize its efficiency during training.

### 2.4. Image Enhancement

Traditional image enhancement methods are typically divided into three categories: spatial domain, frequency domain, and color space enhancement. Spatial domain approaches directly manipulate pixel intensity distributions, offering high real-time performance and suitability for resource-limited environments [32]. Frequency domain techniques transform images to the frequency space and selectively enhance or suppress components to remove noise, particularly effective for mitigating high-frequency noise in low-light conditions [33]. Color space methods adjust saturation, hue balance, and inter-channel correlations to correct distortions and improve signal-to-noise ratio via multi-channel fusion [34]. Despite their effectiveness, these methods struggle with fundamental issues such as underexposure and noise amplification, while over-enhancement can cause color distortion, and repeated color space conversions may introduce computational overhead. Retinex theory has inspired numerous learning-based low-light enhancement models. URetinexNet [35] adopts data-driven unfolding optimization with implicit prior regularization to replace handcrafted decomposition, enabling more adaptive illumination adjustment. Retinexformer [36] utilizes a one-stage illumination-guided framework and non-local attention for global brightness modeling but suffers from high computational cost and limited interpretability. RetinexMamba [37] addresses this by replacing self-attention with a state space model and incorporating fused attention, enhancing both efficiency and robustness.

Generative models have also been extensively applied to low-light enhancement. EnlightenGAN [38] leverages dual discriminators and perceptual self-regularization to improve key region recovery. PyDiff [39] introduces a multi-resolution pyramid diffusion network with adaptive correction, achieving notable PSNR gains. LLFlow [40] employs invertible mappings and normalized flow to model non-deterministic transitions between low-light and normal-light domains. Huang et al. [41] integrated variational autoencoders with Bayesian neural networks, using variational free energy loss and dynamic priors to produce reliable low-light image generation.

Despite significant progress in both traditional and learning-based approaches, achieving robust and efficient enhancement under extreme low-light and complex conditions remains a challenging task.

## 3. Method

**Figure 1:**
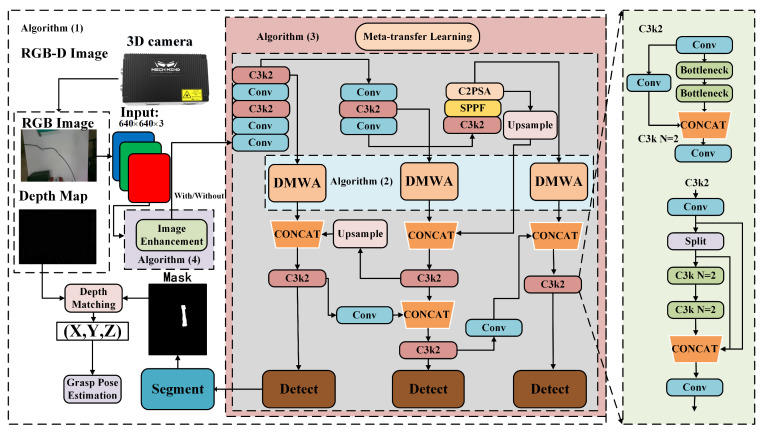
Neural network workflow and the process of combining a 2D segmentation mask with a depth map to obtain 3D coordinates and pose estimation. The white background denotes the overall framework (Algorithm 1), the light blue background represents the DMWA mechanism (Algorithm 2), the light red background indicates the transfer learning framework (Algorithm 3), the light purple background corresponds to the image enhancement module (Algorithm 4), and the gray background corresponds to the visual detection and segmentation module.

The workflow of the proposed framework is outlined in Algorithm 1. The system comprises four core modules: image enhancement, visual detection and segmentation, transfer learning, and attention mechanism. The image enhancement module improves image quality under low-illumination conditions, the visual detection and segmentation module enables accurate extraction of key targets and image-level understanding, the transfer learning module enhances the model’s generalization ability across diverse scenarios, and the attention mechanism module further emphasizes critical features while suppressing redundant background information. Finally, precise alignment between the detection results and 3D point clouds is achieved through point cloud matching, forming an intelligent broken wire detection and segmentation workflow for substations. This section focuses on the proposed attention mechanism module, transfer learning module, and image enhancement approach. The overall process is illustrated in Figure 1.

**Algorithm 1** Generalized broken wire segmentation framework in substation scenarios**Require:** RGB image $I_{RGB}\in R^{m\times n\times 3}$, depth image $D\in R^{m\times n}$, illumination L∈R (in lux), 3D point cloud $P\subset R^{3}$, and a transfer learning model $M_{TL}$ (e.g., pre-trained, meta-transfer, etc.).**Ensure:** Matched point cloud $P_{\mathrm{matched}}\subset R^{3}$  1: Step 1: Image Enhancement$$I_{RGB}^{\prime }=f_{e}\left(I_{RGB},L\right)=\left\{\begin{array}{cc} I_{RGB}, & \mathrm{if}\;400\leq L_{\mathrm{lux}}<600\;\left(\mathrm{Indoor}\right),\\ \mathrm{Enhance}\left(I_{RGB}\right), & \mathrm{otherwise}. \end{array}\right.$$  2:                  $D^{\prime }\leftarrow D$            //Depth image remains unchanged  3: Step 2: Detection and Segmentation via Transfer Learning          Decompose  $M_{TL}$ into Training, as shown in Algorithm 2:$$\phi :R^{m\times n\times 3}\to R^{d},\;\mathrm{feature}\;\mathrm{extractor}$$$$\psi :R^{d}\times R^{m\times n}\to R^{4}\times R^{m\times n},\;\mathrm{detection}\;\mathrm{and}\;\mathrm{segmentation}\;\mathrm{mapper}$$//ψ takes the feature vector from ϕ ($\in R^{d}$) and the depth image ($\in R^{m\times n}$). It outputs a bounding box with 4 parameters (in $R^{4}$, e.g., (x,y,w,h)) and a segmentation mask (in $R^{m\times n}$), fulfilling the detection and segmentation tasks. 4: Compute features from enhanced RGB image:$$z=\phi (I_{RGB}^{\prime })$$ 5: Obtain detection and segmentation results:$$\left(B,S\right)=\psi \left(z,D^{\prime }\right)$$ 6: Step 3: Point Cloud Matching$$P_{\mathrm{matched}}=f_{m}\left(B,S,P\right),\;P_{\mathrm{matched}}\subset P.$$ 7: **return** 
$P_{\mathrm{matched}}$


**Algorithm 2** Dynamic multi-window attention (DMWA)**Require:** Feature map $X\in R^{b\times c\times h\times w}$, Window sizes $\left\{w_{1},w_{2},\ldots ,w_{K}\right\}$, Attention heads $N_{h}$**Ensure:** Enhanced feature map $X_{\mathrm{out}}\in R^{b\times c\times h\times w}$  1: Compute Adaptive Window Weights α using GAP and a 1 × 1 convolution (as Equation (3))  2: Compute Multi-Window Attention for Each $w_{k}$  3: **for**
k=1 to *K* **do**  4:        Partition *X* into non-overlapping windows of size $w_{k}\times w_{k}$  5:        Compute attention $A_{k}$ using multi-head attention (as Equation (4))  6: **end for**  7: Gated Fusion of Multi-Scale Attention to obtain $X_{\mathrm{out}}$ (as Equation (8))  8: Apply window-wise weights α to modulate fusion output  9: **Parameter Optimization**  10: Compute total loss L (as Equations (9)–(12))  11: Update parameters: $\theta \leftarrow \theta -\eta \nabla_{\theta }L$  12: **return** 
$X_{\mathrm{out}}$


**Algorithm 3** Meta-training algorithm  1: **Input:** Support set $S=\{\left(X_{\mathrm{support}},Y_{\mathrm{support}}\right)\}$, Query set $Q=\{\left(X_{\mathrm{query}},Y_{\mathrm{query}}\right)\}$, Learning rates α,β, Regularization parameter λ  2: **Output:** Updated model parameters $\phi^{*},\Phi^{*}$ and meta-loss $L_{\mathrm{meta}}$  3: Initialize model parameters ϕ,Φ  4: **for** each task $T_{i}$ in support set *S* **do**  5:        Support Set Update:  6:        Compute support loss: $L_{s}\left(\Phi ,\phi \right)$; Compute gradient: $\nabla_{\phi }L_{s}\left(\Phi ,\phi \right)$  7:        Update ϕ via Equation (18): $\phi \leftarrow \phi -\alpha \nabla_{\phi }L_{s}\left(\Phi ,\phi \right)$  8: **end for**  9: **for** each task $T_{i}$ in query set *Q* **do**  10:        Query Set Update:  11:        Compute query loss: $L_{q}\left(\Phi ,\phi \right)$; Compute gradient: $\nabla_{\Phi }L_{q}\left(\Phi ,\phi_{i}\right)$  12:        Update Φ via Equation (18): $\Phi \leftarrow \Phi -\beta \sum_{i}\nabla_{\Phi }L_{q}\left(\Phi ,\phi_{i}\right)$  13: **end for**  14: **Total Loss Computation:** Initialize $L_{\mathrm{meta}}\leftarrow 0$  15: **for** each task $T_{i}$ in query set *Q* **do**  16:        Compute query loss: $L_{q}\left(\Phi ,\phi^{\prime }\right)$  17:        Accumulate: $L_{\mathrm{meta}}\leftarrow L_{\mathrm{meta}}+L_{q}\left(\Phi ,\phi^{\prime }\right)$  18: **end for**  19: Compute regularization term via Equation (10):     $L_{\mathrm{reg}}=\lambda {∥A_{\mathrm{DMWA}}^{\left(1\right)}-A_{\mathrm{DMWA}}^{\left(L-1\right)}∥}_{2}^{2}$  20: Compute total meta-loss via Equation (19):     $L_{\mathrm{meta}}\leftarrow L_{\mathrm{meta}}+L_{\mathrm{reg}}$ 21: **Return:** Updated parameters ϕ,Φ, and meta-loss $L_{\mathrm{meta}}$


**Algorithm 4** LP-MSR: Light Prior-Based Multi-Scale Retinex with color restoration**Require:** Input image $I\in R^{H\times W\times 3}$, illumination prior $L_{p}\in R^{H\times W\times 1}$**Ensure:** Enhanced image $I_{\mathrm{enhanced}}\in R^{H\times W\times 3}$  1: Step 1: Concatenate Input with Light Prior  2: Concatenate *I* and $L_{p}$ along the channel axis: $I_{c}\leftarrow \mathrm{concat}\left(I,L_{p}\right)$  3: Step 2: Light Map Generation  4: Extract features: $x\leftarrow {\mathrm{Conv}}_{1\times 1}\left(I_{c}\right)$  5: Local feature extraction: $F_{lu}\leftarrow {\mathrm{DepthwiseConv}}_{5\times 5}\left(x\right)$  6: Light map: $L\leftarrow {\mathrm{Conv}}_{1\times 1}\left(F_{lu}\right)$  7: Step 3: Multi-Scale Retinex with Color Restoration   8: **for** each $\sigma \in \{\sigma_{1},\sigma_{2},\sigma_{3}\}$ **do**  9:         $B_{\sigma }\leftarrow \mathrm{GaussianBlur}\left(I,\sigma \right)$  10:       $R_{\sigma }\leftarrow \log\left(I+1\right)-\log\left(B_{\sigma }+1\right)$  11: **end for**  12:
$R\leftarrow \frac{1}{3}\sum_{\sigma }R_{\sigma }$  13: Color restoration:  14:
$C\left(h,w,c\right)=\log\left(\alpha \cdot I\left(h,w,c\right)+1\right)-\log\left(\sum_{c^{\prime }}I\left(h,w,c^{\prime }\right)+1\right)$  15: Final Retinex output: $I_{\mathrm{msrcr}}=G\cdot R\cdot C+b$  16: Step 4: Final Enhancement  17: 
$I_{\mathrm{enhanced}}\leftarrow I_{\mathrm{msrcr}}+L$                **return** 
$I_{\mathrm{enhanced}}$


### 3.1. Dynamic Multi-Window Attention

**Figure 2:**
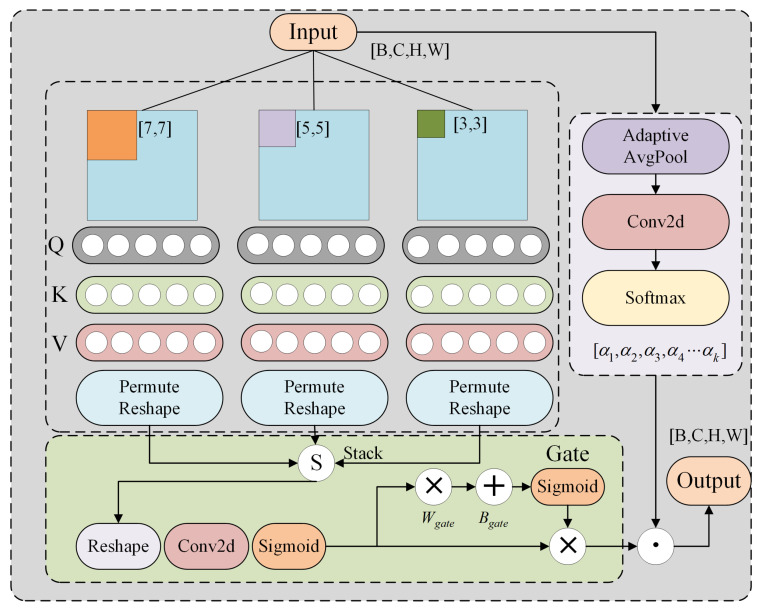
Architectural details of the DMWA module. The green regions represent the gating mechanisms, while the light purple areas indicate adaptive weights.

W-MSA employs a single fixed-size window, which limits its adaptability to varying spatial patterns. Although SW-MSA [42] introduces shifted windows to alleviate locality constraints, it still lacks sufficient capability in capturing multi-scale information. To address this, we propose a dynamic multi-scale window attention (DMWA) mechanism that integrates multi-scale window compositions with adaptively learned weights to improve cross-scale feature fusion, as illustrated in Figure 2.

Let the input feature map be denoted as $X\in R^{H\times W\times C}$, where *H* and *W* represent the spatial dimensions, and *C* is the number of channels. We define a set of candidate window sizes as $M=\{m_{1},m_{2},\ldots ,m_{k}\}$, for example, M={3,5,7}. A global feature representation is used to generate attention weights $\alpha \in R^{k}$, which satisfy the constraint $\sum_{i=1}^{k}\alpha_{i}=1$. For each window size $m_{i}\in M$, the input feature map is partitioned into regular non-overlapping grids: the input feature map is first padded to match the partition size for each window scale, as defined in Equation (1).

(1) $$X_{\mathrm{pad}}^{i}=RP\left(X,m_{i}\right)$$

Here, RP denotes reflection padding, which resizes the input feature map to a padded size of $H^{\prime }\times W^{\prime }$, where $H^{\prime }\;\mathrm{mod}\;m_{i}=0$ and $W^{\prime }\;\mathrm{mod}\;m_{i}=0$.

(2) $$P_{i}\left(X\right)\in R^{\frac{H^{\prime }}{m_{i}}\times \frac{W^{\prime }}{m_{i}}\times m_{i}\times m_{i}\times C}$$

Here, $\frac{H^{\prime }}{m_{i}}\times \frac{W^{\prime }}{m_{i}}$ represents the number of window grids, $m_{i}\times m_{i}$ denotes the spatial size of each window, and *C* is the number of channels. The window weights are generated through global average pooling and learnable parameters as follows:

(3) $$\alpha =\mathrm{Softmax}\left(W_{\alpha }\cdot \mathrm{GAP}\left(X\right)\right),\;W_{\alpha }\in R^{k\times C}$$

where GAP refers to global average pooling, $\mathrm{GAP}\left(X\right)=\frac{1}{HW}\sum_{h=1}^{H}\sum_{w=1}^{W}X_{h,w,:}\in R^{C}$. For each window size $m_{i}$, multi-head self-attention is computed, as follows:

(4) $${\mathrm{Attention}}_{i}\left(Q_{i},K_{i},V_{i}\right)=\mathrm{Softmax}\left(\frac{Q_{i}K_{i}^{T}}{\sqrt{d_{k}}}+B_{i}\right)V_{i}$$

where $Q_{i}=P_{i}\left(X\right)\cdot W_{Q}^{i}$, $K_{i}=P_{i}\left(X\right)\cdot W_{K}^{i}$, and $V_{i}=P_{i}\left(X\right)\cdot W_{V}^{i}$. The projection weights are $W_{Q}^{i},W_{K}^{i},W_{V}^{i}\in R^{C\times d_{k}}$, and $d_{k}=C/h$, where *h* is the number of attention heads. $B_{i}\in R^{m_{i}^{2}\times m_{i}^{2}}$ denotes the relative position encoding matrix, which is generated via learnable parameters. We define the pixel offset within a window as $\Delta =(\delta_{h},\delta_{w})$. The relative position bias matrix $B_{i}$ is obtained via embedding lookup, with the index calculated as follows:

(5) $$\mathrm{Index}\left(\Delta \right)=\delta_{h}\cdot \left(2m_{i}-1\right)+\delta_{w}$$

(6) $$B_{i}=\mathrm{Embedding}\left(\mathrm{Index}\left(\Delta \right)\right)\in R^{m_{i}^{2}\times m_{i}^{2}}$$

The attention output for each window size $m_{i}$ is denoted as $F_{i}\in R^{H\times W\times C}$, and the gating map $g\in R^{H\times W\times k}$ is generated via the following:

(7) $$g=\sigma ({\mathrm{Conv}}_{1\times 1}\left(\mathrm{Concat}\left(F_{1},F_{2},\ldots ,F_{k}\right)\right))$$

where σ is the Sigmoid function. The final output is obtained by performing a weighted combination and separation of multi-scale features.

Here, σ denotes the Sigmoid function, and $g\in R^{H\times W\times k}$. The final output is the weighted combination of multi-scale features, and the segmentation loss is defined as follows:

(8) $$F_{\mathrm{out}}=\sum_{i=1}^{k}\alpha_{i}\cdot \left(g_{i}⊙F_{i}\right)$$

(9) $$L_{\mathrm{dice}}=1-\frac{2\sum_{h,w}Y_{\mathrm{true}}\cdot Y_{\mathrm{pred}}+\varepsilon }{\sum_{h,w}Y_{\mathrm{true}}+\sum_{h,w}Y_{\mathrm{pred}}+\varepsilon }$$

Class imbalance is common in industrial scenarios, especially when detecting small or damaged regions in power lines. In hierarchical prediction tasks, when predicted probabilities approach 0 or 1, model sensitivity decreases, potentially causing overconfidence. We introduce the following attention smoothness regularization term:

(10) $$L_{\mathrm{reg}}=\sum_{l=1}^{L}{∥A^{\left(l\right)}-A^{\left(l-1\right)}∥}_{F}^{2}$$

(11) $$∥A^{\left(l\right)}-A^{\left(l-1\right)}{∥}_{F}=\sqrt{\sum_{i,j}{\left(A_{i,j}^{\left(l\right)}-A_{i,j}^{\left(l-1\right)}\right)}^{2}}$$

$A^{\left(l\right)}$ denotes the attention map at layer *l*, computed from the attention score matrix $QK^{T}$.The overall loss is given by the following:

(12) $$L=L_{\mathrm{dice}}+\lambda L_{\mathrm{reg}}$$

The receptive field of traditional convolution is defined as $R_{\mathrm{conv}}={\left(k+\left(s-1\right)\left(d-1\right)\right)}^{2}$, while the receptive field of DMWA is defined as follows:

(13) $$R_{\mathrm{DMWA}}=\overset{k}{\underset{i=1}{⋃}}{\left[0,m_{i}+2^{l-1}\right]}^{2}$$

Let the mutual information between input *X* and label *Y* be I(X;Y); then DMWA improves information retention through multi-scale integration, as follows:

(14) $$I_{\mathrm{DMWA}}\left(X;Y\right)=I_{\mathrm{base}}\left(X;Y\right)+\sum_{i=1}^{k}\alpha_{i}I\left(F_{i};Y|X\right)$$

If the set $\left\{F_{i}\right\}$ is conditionally independent, mutual information increases linearly. Under gradient descent with the learning rate η, the convergence rate satisfies the following:

(15) $$E\left[L\left(t+1\right)\right]\leq L\left(t\right)-\eta \nabla L^{2}+\frac{\eta^{2}L}{2}\left(\sigma^{2}+\lambda^{2}k^{2}\varepsilon^{2}\right)$$

The regularization term $L_{\mathrm{reg}}$ thus helps suppress gradient explosion and improve convergence. The overall process is detailed in Algorithm 2.

### 3.2. Multi-Stage Meta-Transfer Learning

Due to the limited availability of training samples, conventional deep learning methods struggle to effectively learn features from small-scale damaged regions. Traditional transfer learning approaches often transfer all knowledge from the source domain to the target domain, which may result in negative transfer. Although meta-transfer learning can mitigate some negative transfer effects, it still suffers from performance degradation when the domain gap between source and target is too large.

**Figure 3:**
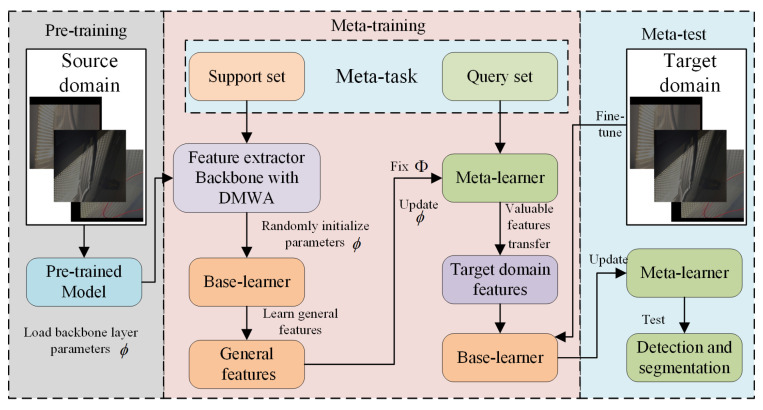
Workflow of the meta-transfer learning.

To address these limitations, we propose a multi-stage meta-transfer learning strategy that selectively freezes different network layers at different training stages to better adapt to the challenges caused by variations in lighting and complex backgrounds. The learning process is divided into three stages: (a) pre-training, (b) meta-training, and (c) meta-testing, as illustrated in Figure 3.

#### 3.2.1. Pre-Training

In the pre-training stage, the pretrained weights of YOLOv11 are fine-tuned using a damaged conductor dataset under normal illumination from the source domain $D_{s}$. Both the Backbone and the Neck layers are used as the feature extractor Φ. The cross-entropy loss L is employed to update Φ, defined as follows:

(16) $$L\left(\Phi \right)=-\frac{1}{D_{s}}\left[Y\log(p(x))+(1-Y)\log(1-p(x))\right]$$

(17) $$\Phi =\Phi -\alpha \nabla_{\Phi }L\left(\Phi \right)$$

where α denotes the learning rate during pre-training. The feature extractor Φ obtained from this step is then transferred into the meta-training phase to enhance the robustness and convergence speed of the proposed framework.

#### 3.2.2. Meta-Training

During meta-training, a meta-task T∼p(T) is constructed by randomly sampling from the source domain $D_{s}$. Each meta-task consists of two subsets: a support set $D_{s}^{s}$ and a query set $D_{s}^{q}$. The support set $D_{s}^{s}$ is used to update the base learner, while the query set $D_{s}^{q}$ is used to evaluate and further adapt the learner.

Specifically, for each meta-batch, the base learner is initialized with the pre-trained feature extractor parameters from YOLOv11 and a random initialization of the remaining parameters. Each meta-batch contains a batch of tasks, and each task has its own base learner. Using gradient descent, the base learner is updated to learn generic features of damaged conductors, as shown below:

(18) $$\phi^{*}=\phi -\alpha \cdot \nabla_{\phi }L_{T_{i}}\left(f_{\phi }\right),\;\Phi^{*}=\Phi -\alpha \cdot \nabla_{\Phi }L_{T_{i}}\left(f_{\Phi }\right)$$

Here, $\phi^{*}$ and $\Phi^{*}$ are the updated parameters of the base learner on the support and query sets, respectively; α is the learning rate, and $L_{T_{i}}\left(f_{\phi }\right)$, $L_{T_{i}}\left(f_{\Phi }\right)$ denote the task-specific cross-entropy loss.

Conventional meta-transfer learning focuses on optimizing the entire network using fast adaptation of initial parameters. However, transferring the entire network in the meta-training stage may result in overfitting, especially in early convolutional layers. To avoid this, we freeze the pre-trained and fine-tuned shallow layers of the network and only perform meta-transfer on the Backbone, DMWA, and Neck modules. This improves training efficiency and convergence speed. The meta-training loss is formulated as follows:

(19) $$L_{\mathrm{meta}}=\sum_{i}L_{\mathrm{query}}\left(\Phi ,\phi_{\mathrm{DMWA}}^{\prime },\phi_{\mathrm{Neck}}^{\prime }\right)+\lambda {∥A_{\mathrm{DMWA}}^{\left(l\right)}-A_{\mathrm{DMWA}}^{\left(l-1\right)}∥}_{F}^{2}$$

where $L_{\mathrm{query}}$ is the cross-entropy loss on the query set, and $\phi_{\mathrm{DMWA}}^{\prime }$, $\phi_{\mathrm{Neck}}^{\prime }$ are the updated parameters of DMWA and Neck. The regularization term compares the attention maps of the first and last layers in DMWA—$A_{\mathrm{DMWA}}^{\left(l\right)}$ and $A_{\mathrm{DMWA}}^{\left(l-1\right)}$—and smooths their difference to ensure stability during meta-learning.The training details of meta-training are described in Algorithm 3.

#### 3.2.3. Meta-Test

During meta-testing, a set of tasks ${\left\{T_{s}\right\}}_{1}^{N}$ is sampled from the target domain $D_{T}$ under various illumination conditions. These tasks are similarly split into a support set $D_{T}^{s}$ and a query set $D_{T}^{q}$. The optimized parameters $\phi^{*}$ and $\Phi^{*}$ obtained from meta-training are used to initialize the model for meta-testing. The base learner is further fine-tuned on the support set $D_{T}^{s}$ to adapt to the target domain.

Since the previous two stages were conducted on the source domain $D_{S}$ (collected under normal illumination), the meta-testing phase requires the model to adapt to feature distribution shifts caused by different lighting conditions. To this end, the Backbone is frozen during this phase, and a small learning rate (e.g., $1\times 10^{-5}$) is used to fine-tune all other network layers. The final optimized parameters $\phi^{*}$ and $\Phi^{*}$ are then used for evaluation.

### 3.3. Low-Light Image Enhancement

In low-light environments, both global contrast and local noise can adversely affect object detection and semantic segmentation. However, their relative impact often depends on specific tasks and environmental conditions. Generally, global contrast plays a more critical role than local noise, particularly under poor illumination. Images with low global contrast typically lack essential semantic cues across the entire scene, making it difficult to distinguish objects from the background. This significantly degrades segmentation accuracy, especially when the contrast is insufficient for object delineation. Under such conditions, the model struggles to detect or accurately segment objects.

In contrast, local noise tends to affect only small regions, primarily degrading boundary quality. Its impact is often mitigated by smoothing operations or specific architectural designs, such as using dilated convolutions or edge-aware modules.

Inspired by Retinex theory and considering the limited computational resources in industrial applications—which constrain the use of overly deep enhancement networks—we introduce an efficient enhancement method that combines illumination prior compensation with color image enhancement. This approach focuses on improving global contrast using minimal computation. The proposed method, LP-MSR (Light Prior-based Multi-Scale Retinex with Color Restoration), is summarized in Algorithm 4.

**Figure 4:**
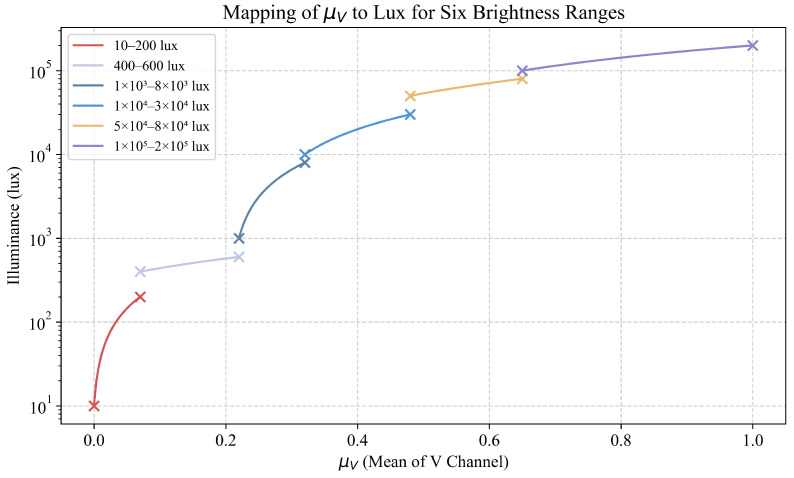
Mapping of $\mu_{V}$ to lux for six brightness ranges.

In addition, LP-MSR employs a single-pass computation of the image’s mean luminance (HSV–V channel, Hue–Saturation–Value) as a proxy for ambient lux, using a precomputed calibration curve to map $\mu_{V}$ to approximate lux and thus assign one of six brightness categories (shown in Table 1). Specifically, let

(20) $$\mu_{V}\;=\;\frac{1}{H\times W}\sum_{h=1}^{H}\sum_{w=1}^{W}V\left(h,w\right)$$

where V(h,w) is the value channel at the pixel (h,w), *H* and *W* represent the height and width of the image, respectively. The *V* channel in the HSV color model indicates the brightness or lightness of a color, with higher values corresponding to brighter colors. The *V* channel, along with the hue (*H*) and saturation (*S*) channels, is used to describe the full color representation of each pixel in the image. We then apply a calibration function $L_{\mathrm{lux}}=f\left(\mu_{V}\right)$ (obtained by collecting data from a predefined dataset under the six initial scenes, with the distribution of the *V* channel estimated from the readings under known illuminance conditions, as measured by a calibrated lux meter) to estimate lux, as shown in Figure 4. The six estimated lux ranges are as follows:

(21) $$L_{\mathrm{lux}}=\left\{\begin{array}{cc} \mathrm{Range}\;1:\;L_{\mathrm{lux}}<200, & (\mathrm{Low}\;\mathrm{Light})\\ \mathrm{Range}\;2:\;400\leq L_{\mathrm{lux}}<600, & \left(\mathrm{Indoor}\right)\\ \mathrm{Range}\;3:\;1000\leq L_{\mathrm{lux}}<8000, & (\mathrm{Weak}\;\mathrm{Cloudy}\;\mathrm{Light})\\ \mathrm{Range}\;4:\;10000\leq L_{\mathrm{lux}}<30000, & (\mathrm{Strong}\;\mathrm{Cloudy}\;\mathrm{Light})\\ \mathrm{Range}\;5:\;50000\leq L_{\mathrm{lux}}<80000, & (\mathrm{Clear}\;\mathrm{Strong}\;\mathrm{Light})\\ \mathrm{Range}\;6:\;L_{\mathrm{lux}}\geq 100000, & (\mathrm{Extreme}\;\mathrm{Strong}\;\mathrm{Light}) \end{array}\right.$$

For images in Range 1, we increase the color-restoration coefficient α and Retinex gain *G*, while reducing each Gaussian scale $\sigma_{i}$ to emphasize detail recovery, as described in lines 8–15 of Algorithm 4. For Range 2, no enhancement is applied (all parameters remain at their defaults). For Range 6, α and *G* are reduced and $\sigma_{i}$ is increased to prevent over-enhancement. For the intermediate ranges (3–5), all parameters interpolate linearly between their low- and high-lux settings. This one-time O(HW) luminance scan, calibration lookup, and linear interpolation incur negligible overhead compared with the multi-scale Gaussian blurs and convolutions, leaving inference complexity effectively unchanged.

## 4. Experiments and Discussion

### 4.1. Evaluation Indicators

In object detection and segmentation tasks, commonly used evaluation metrics include recall, precision, average precision (AP), and mean average precision (mAP). Recall measures the proportion of actual positive samples that are correctly identified, while precision indicates the proportion of detected positive samples that are true positives. These metrics often have a trade-off: increasing recall may decrease precision due to false positives, while increasing precision may decrease recall, leading to missed detections. The formulas for these metrics are as follows:

(22) $$\mathrm{Recall}=\frac{TP}{TP+FN},\;\mathrm{Precision}=\frac{TP}{TP+FP}$$

where TP is the number of true positives, FN is the number of false negatives, and FP is the number of false positives.

(23) $$AP=\int_{0}^{1}\mathrm{Precision}\left(r\right)\;dr,\;mAP=\frac{1}{C}\sum_{i=1}^{C}AP_{i}$$

where Precision(r) is the precision at each recall level *r*, *C* is the number of classes, and $AP_{i}$ is the average precision for the *i*-th class.

Additionally, FPS (frames per second) measures the real-time processing capability of the model, indicating how many images the model can process per second. GFLOPs (Giga Floating Point Operations per Second) indicates the amount of computation required for a single forward pass through the model, and Parameters represents the total number of trainable parameters in the model.

(24) $$FPS=\frac{\mathrm{Number}\;\mathrm{of}\;\mathrm{frames}\;\mathrm{processed}}{\mathrm{Time}\;\mathrm{taken}\;\mathrm{to}\;\mathrm{process}\;\mathrm{the}\;\mathrm{frames}}$$

(25) $$\mathrm{GFLOPs}=\frac{\mathrm{Total}\;\mathrm{number}\;\mathrm{of}\;\mathrm{floating}\;\mathrm{point}\;\mathrm{operations}}{10^{9}}$$

These metrics provide insights into both the performance and computational efficiency of the model.

### 4.2. Experiment Setup

To train and validate the performance of the damaged wire detection and segmentation model in substations, this study constructed two datasets, with a particular focus on the impact of lighting variations on model performance.

1. Complex Background Dataset: The complex background dataset comprises 3760 RGB images encompassing various wire forms and damage types, making it well-suited for complex substation environments. Data augmentation techniques—including rotation, scaling, brightness adjustment, and noise injection—were applied to enhance the model’s robustness under diverse environmental conditions.
2. Lighting Variation Test Datasets: The lighting variation test datasets consist of 1350 images designed to evaluate model performance under a range of lighting conditions. The light intensity spans from 10 to 200,000 lux, covering scenarios such as nighttime low light, typical outdoor weak light, direct strong sunlight, and extreme brightness. Details of the lighting conditions are summarized in Table 1.

In the initial stage, the batch size was set to 16, and the model was trained for 50 epochs with a 20-epoch warm-up strategy. The initial learning rate was set to 0.01. The optimizer used was Adam, with parameters $\beta_{1}=0.9$, $\beta_{2}=0.999$, and a weight decay of $5\times 10^{-4}$. Based on this setup, we performed grid search to determine the optimal learning rate from the candidate set $\left\{1\times 10^{-2},5\times 10^{-3},1\times 10^{-3}\right\}$, combined with data augmentation probabilities from {0,0.5,1.0}.

After evaluating the mean Average Precision (mAP) on the complex background and lighting variation test datasets, experimental results showed that a learning rate of $5\times 10^{-3}$, together with Mosaic = 1.0 and Mixup = 0.15, enabled fast convergence and reduced the risk of overfitting. Subsequently, we extended the training to 150 epochs and adopted multi-scale training, where the input image size was randomly resized within the range [320,640] to improve the model’s adaptability to different target scales. The hardware configuration used for training is shown in Table 2.

**Figure 5:**
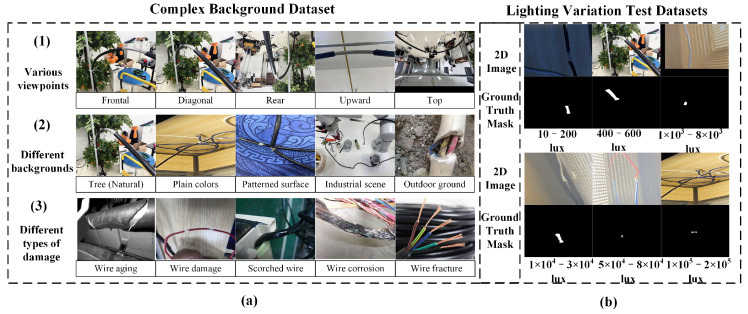
Classification of datasets. (**a**) Represents the complex background dataset, collected with variations across three aspects: different viewpoints (frontal, diagonal, rear, upward, top), different backgrounds (tree/natural, plain colors, patterned surface, industrial scene, outdoor ground), and different types of damage (wire aging, wire damage, scorched wire, wire corrosion, wire fracture). (**b**) Represents the test datasets under six different lighting conditions, with lighting variations ranging from 10 to 2 × 10^5^ lux, as defined in Table 1. The binary image shown in (**b**) corresponds to the ground truth (GT) mask.

As shown in Figure 5, the complex background dataset was collected as illustrated in Figure 5a. Images were captured from various perspectives, including frontal, diagonal, rear, upward, and top–down views, providing a panoramic representation of the operational environment. The dataset includes wires placed against diverse backgrounds, such as trees, flat surfaces, industrial areas, and open ground, and under varying levels of occlusion. Each wire instance exhibits different damage states, including aging, deformation, scorching, corrosion, and fracture. Additionally, as shown in Figure 5b, the Lighting Variation Test Datasets were captured under six distinct lighting conditions to simulate a wide range of real-world scenarios: Low Light Conditions, Indoor Lighting, Overcast Weak Light, Overcast Strong Light, Cloudless Strong Light, and Extreme Strong Light.

To ensure the accuracy of the lighting variation test set, we controlled the distance between the light source and the object and used a photometer to measure the range of illumination intensity. The control of illumination intensity was achieved using the following formulas. The expressions of illumination calculation based on luminous flux and inverse-square law are shown in the following Equations (26) and (27):

(26) $$E=\frac{\Phi }{A}$$

(27) $$E=\frac{I}{d^{2}}$$

Here, *E* is the illuminance, measured in lux; Φ is the luminous flux, measured in lumens (lm); *A* is the illuminated area, measured in square meters (m^2^); *I* is the luminous intensity, measured in candelas (cd); and *d* is the distance between the light source and the object, measured in meters (m).

To derive Equation (28), we combine the concepts of image brightness, light source distribution, and object reflectance characteristics, as follows:

(28) $$I\left(x,y\right)=\int_{0}^{\infty }L\left(x,y,z\right)R\left(x,y,z\right)\;dz=\int_{0}^{\infty }\frac{\Phi \cdot R(x,y,z)}{A\cdot d^{2}}\;dz$$

Here, I(x,y) represents the image brightness, L(x,y,z) denotes the brightness distribution of the light source, and R(x,y,z) represents the reflectance properties of the object.

### 4.3. Result Analysis

#### 4.3.1. DMWA Module Effectiveness Verification

To evaluate the effectiveness of the proposed attention mechanism, we replaced the original attention module in our framework with several commonly used alternatives. Experiments were conducted on our normal-light dataset (400–600 lux) and on the complex background dataset, where we compared W-MSA [42], SW-MSA [42], SE [43], CBAM [44], ECA [45], and SRM [46]. The results are summarized in Table 3 and Table 4.

As shown in Table 3 and Table 4, on the normal-light dataset (400–600 lux), our proposed DMWA achieves the largest gains across four key metrics: detection accuracy (+14.99%), detection F1-Score (+13.54%), segmentation accuracy (+13.02%), and segmentation F1-Score (+11.06%). These improvements significantly outperform all competing methods. Notably, on the complex background dataset, SRM’s performance increases slightly compared with the normal-light dataset (detection accuracy: +3.11% to +4.14%, detection F1: +4.22% to +5.13%; segmentation accuracy: +4.78% to +5.07%). In contrast, other attention modules see drops in their metrics under complex backgrounds, yet DMWA still delivers superior improvements over all other attention mechanisms.

Moreover, DMWA also demonstrates strong practical performance in terms of inference speed, achieving 47.9 FPS and an inference latency of only 1.5 ms. Compared with sliding-window-based mechanisms, DMWA achieves a better trade-off between accuracy and efficiency. While both W-MSA and SW-MSA yield competitive accuracy, they fall short in inference speed, highlighting the superior balance offered by DMWA.

#### 4.3.2. Multi-Stage Meta-Transfer Learning Effectiveness Verification

We adopt different transfer learning strategies to train the visual module, including Transfer Learning (TL) [24] and Meta-Transfer Learning (MTL) [47], with the following configurations:

- TL: The model is first pretrained on the source domain for 50 epochs, during which the Backbone layers are frozen to preserve feature extraction capabilities and prevent overfitting on limited data. The remaining layers are then fine-tuned for another 100 epochs.
- MTL: Each meta-batch contains 10 meta-tasks. Both the support and query sets follow a five-way five-shot setting and cover six illumination conditions. The learning rate for both the base learner and the meta learner is set to 0.001. The base learner is trained for 10 inner-loop steps, while the meta learner updates once per meta-iteration. The total training comprises 150 epochs.

To enhance learning efficiency under few-shot, multi-illumination, and multi-scene conditions, and to mitigate the issue of negative transfer in conventional methods, we propose a multi-stage meta-transfer learning approach (MMTL). MMTL achieves faster convergence with limited data and significantly improves the F1-Score, thereby enhancing generalization performance.

**Figure 6:**
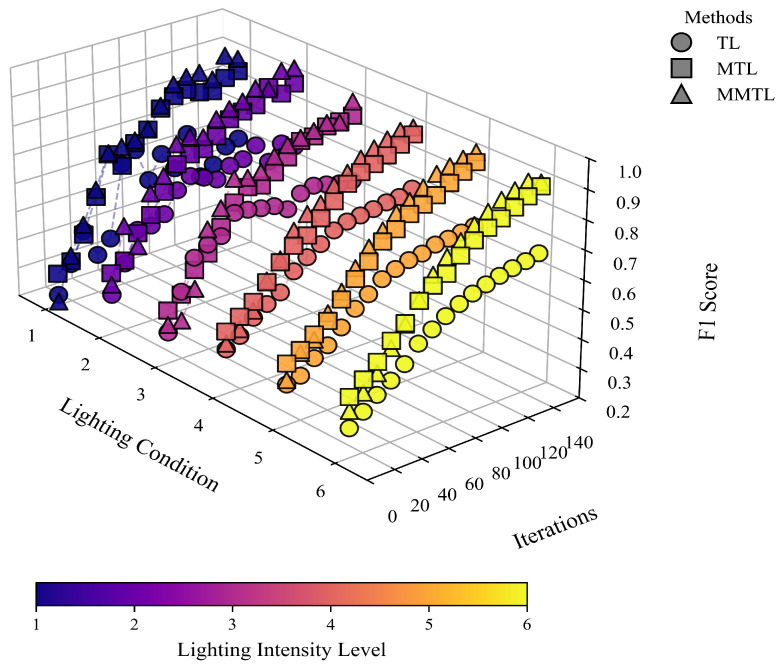
Three-dimensional visualization of F1-Score evolution under different lighting conditions and training methods.

We compare the F1-Scores of TL, MTL, and MMTL after 150 training epochs under various illumination settings (as shown in Figure 6). Experimental results show that TL struggles to exceed an F1-Score of 0.7 in few-shot scenarios and is prone to negative transfer. Although MTL performs better, global fine-tuning can lead to the loss of some generalizable features. In contrast, MMTL integrates meta-learning mechanisms with a staged freezing strategy, effectively avoiding negative transfer and achieving high F1-Scores (up to 0.93) within a shorter training period. These results demonstrate its strong generalization ability and stability in low-data regimes.

#### 4.3.3. Generalization and Robustness Verification of Vision Modules

**Figure 7:**
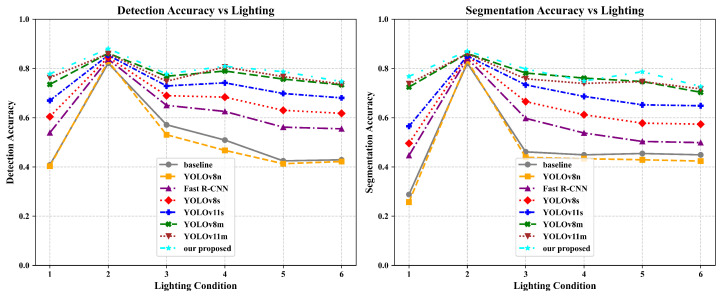
Comparison of detection and segmentation performance of different models under different illuminations. The six lighting conditions (1–6) correspond to those defined in Table 1.

To evaluate the performance and robustness of the proposed visual model Yolov11n: DMWA-MMTL under complex backgrounds and varying lighting conditions, we conducted tests on an illumination-variant dataset, with a focus on detection and segmentation accuracy. The overall performance results are illustrated in Figure 7.

In terms of detection and segmentation accuracy, our method consistently outperforms the YOLO series (e.g., YOLOv8n, YOLOv11s, YOLOv11m) and Fast R-CNN across all lighting scenarios. Under low-light conditions (10–200 lux), our model achieves a detection accuracy of 0.7778, significantly higher than that of Fast R-CNN (0.4032) and the baseline (0.4078). In normal lighting (400–600 lux), it reaches the highest accuracy of 0.8804, surpassing YOLOv11m (0.7628) by approximately 12%. Even under high illumination (1 × 10^5^–2 × 10^5^ lux), the model maintains a strong accuracy of 0.7467, clearly outperforming the baseline (0.4289) and other methods.

A similar trend is observed in segmentation tasks. Our model achieves 0.7678 segmentation accuracy in low-light conditions, far exceeding Fast R-CNN (0.4467), and reaches 0.8704 under standard lighting, outperforming all comparison models. Overall, the proposed method not only achieves higher average accuracy, but also demonstrates greater robustness and stability under extreme illumination conditions.

#### 4.3.4. Validation of the Proposed Image Enhancement Method and Overall Framework

To validate the effectiveness of our proposed image enhancement approach in improving segmentation performance under low-light conditions, we conducted a series of ablation studies by replacing both the visual detection and segmentation modules as well as the image enhancement module. These experiments were performed on a low-light dataset (10–200 lux) to evaluate detection and segmentation capabilities.

To ensure real-time inference on resource-constrained edge devices, this work follows a real-time-first principle and therefore compares only lightweight architectures that have been proven feasible for mobile and embedded scenarios. Deep, high-capacity models such as the UNet family were excluded from our comparative experiments because their higher FLOPs and memory footprint prevent them from achieving over 20 FPS on platforms like Raspberry Pi 4B and Jetson Nano under the low-power and low-latency requirements of our target applications.

As shown in Table 5, using our proposed visual module alone without any enhancement module (Model B) already yields a significant improvement in detection and segmentation performance under low-light conditions compared with the baseline (Model A). However, this comes at a slight cost to real-time performance, with FPS dropping from 128.8 to 47.9. When the proposed image enhancement (IE) module is further incorporated (Model H), both detection and segmentation accuracy are further improved (e.g., detection precision increases from 0.763 to 0.862, and recall improves from 0.791 to 0.881). Meanwhile, the inference speed only slightly decreases to 46.4 FPS, which still meets real-time processing requirements.

**Figure 8:**
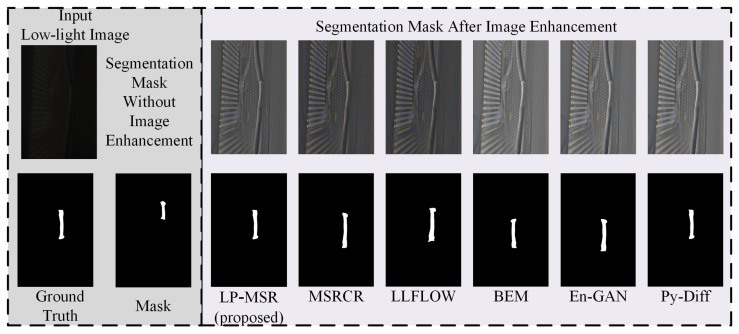
Comparison of low-light image enhancement methods and their segmentation outputs.

Detection and segmentation models with strong generalization capabilities can partially mitigate the accuracy loss caused by noise and reduced clarity. Our image enhancement method places a higher emphasis on improving global contrast, whereas large-scale image enhancement modules invest substantial computational resources into denoising and sharpening. As shown in Figure 8, our shallow LP-MSR outperforms MSRCR, which has similar computational overhead, and achieves performance on par with four deeper image enhancement methods. Moreover, when our enhanced images are used for segmentation, the edges at both ends of damaged conductors are detected more accurately. However, such large network-based enhancement modules are not well suited for industrial applications that demand real-time performance.

Compared with other classical or recent low-light enhancement methods (Models J–M), although they can also significantly boost detection and segmentation performance, most of them suffer from lower speeds (typically under 30 FPS), which limits their real-time applicability. Furthermore, when replacing different detection and segmentation networks (Models N–S), while keeping our IE module unchanged, a good balance between performance and speed can still be achieved. For instance, Model S reaches a detection precision and recall close to 0.86 while maintaining a high speed of 84.7 FPS.

**Figure 9:**
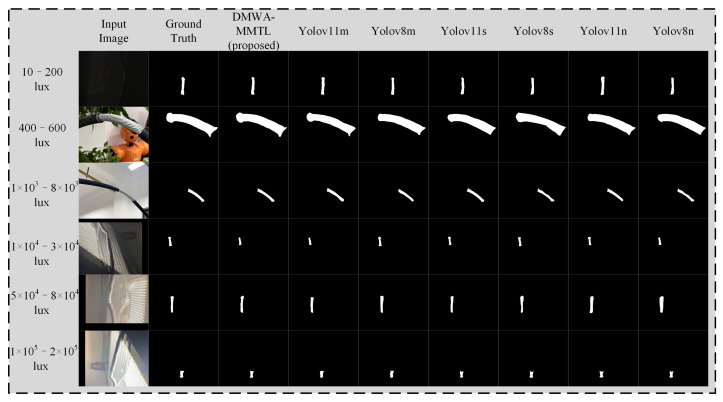
Inference results of seven different models on six different light conditions.

**Figure 10:**
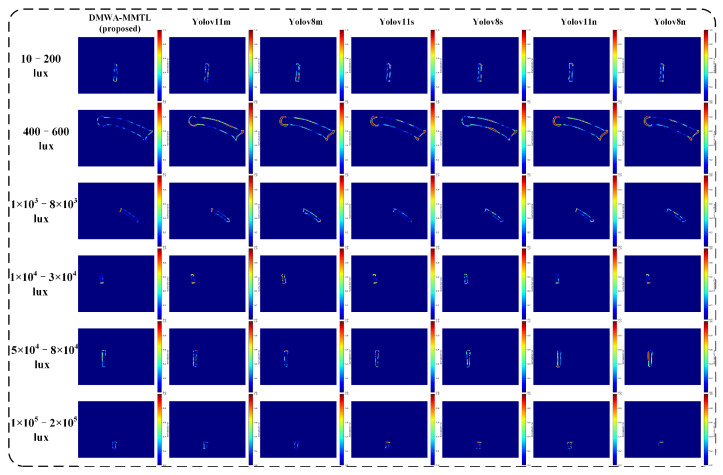
Inference results of seven models in six lighting conditions shown as pixel-level error maps.

From Figure 9 and Figure 10, it is evident that our proposed DMWA-MMTL consistently produces accurate segmentation masks across a wide range of illumination conditions—from extremely low light (10–200 lux) to very high brightness (1 × 10^5^–2 × 10^5^ lux). Unlike the various YOLO-based models, which sometimes lose continuity or misinterpret edges under challenging lighting, our method retains the correct shape and boundary details, closely matching the ground truth. This robustness highlights the strong generalization capability and reliability of our approach, making it particularly suitable for industrial inspection tasks where lighting can vary dramatically.

In summary, the results demonstrate that our proposed image enhancement module can significantly improve detection and segmentation performance in low-light environments while maintaining high real-time performance. These findings further confirm the effectiveness and practicality of the overall framework for low-light vision tasks.

#### 4.3.5. Edge Deployment Considerations

In this section, we choose to conduct our evaluations on the existing experimental equipment available to us and categorize the evaluated platforms into three groups. The first group consists of desktop/laptop-level discrete GPUs—namely, RTX 3090 (24 GB GDDR6X), RTX 1080 (8 GB GDDR5X), and RTX 3060 Laptop (6 GB GDDR6)—which offer very high deep neural network inference throughput, thanks to large numbers of CUDA cores and high-bandwidth VRAM (Video Random Access Memory). The second group comprises ultraportable hybrid-architecture CPUs: Intel Core Ultra 9 (36 MB L3 cache, 6 Performance+Efficient cores) and Core Ultra 7 (24 MB L3 cache, 6 P-Cores+8 E-Cores). Each integrates a Xe iGPU but relies heavily on its large L3 cache and heterogeneous core design to accelerate inference. Finally, the third group includes conventional CPUs—Intel Core i7 (16 MB L3, 6 cores/12 threads) and Core i5 (12 MB L3, 6 cores/12 threads)—which depend solely on FP32 multithreaded execution without heterogeneous cores or dedicated ML accelerators.

As illustrated in Table 6, discrete GPU platforms (RTX 3090/1080/3060 Laptop) run our 17.7 GFLOPs model at 40.1–42.7 FPS versus YOLO11n (6.9 GFLOPs) at 101.9–128.8 FPS, maintaining over 30 FPS even with greater complexity. In the Core Ultra series, large 36 MB/24 MB L3 caches and heterogeneous P/E cores help reduce overhead: our model achieves 26.8 FPS (37.3 ms) on Ultra 9 and 21.3 FPS (46.9 ms) on Ultra 7, compared with YOLO11n’s 52.1 FPS and 39.9 FPS; nonetheless, throughput remains below GPU levels. Conventional CPUs face pronounced cache and memory-bandwidth bottlenecks: Core i7 (16 MB L3) yields only 10.9 FPS (91.7 ms) for our model versus 21.4 FPS for YOLO11n, and Core i5 (12 MB L3) achieves 8.9 FPS (112.4 ms) versus 19.6 FPS, both far below real-time requirements.

Under a strict real-time requirement (≥30 FPS), only discrete GPUs (e.g., RTX 3060 Laptop or higher) can run the FP32 model over 30 FPS without further tuning. Core Ultra 9/Ultra 7 in FP32 yields 26FPS/21FPS and require FP16 or INT8 quantization to exceed 30FPS. Core i7/i5 CPUs cannot reach 30FPS in FP32 (only 8–12FPS), and quantization alone is insufficient.

Under a near-real-time requirement (20 FPS), Core Ultra 9/Ultra 7 in FP32 already meets the threshold (26 FPS/21 FPS). Since NVIDIA’s Jetson Xavier NX edge module offers roughly one quarter of the FP32 throughput of an RTX 3060 Laptop but compensates with Tensor Cores, we estimate that TensorRT FP16/INT8 on Xavier NX yields 20–25 FPS. Similarly, AGX Xavier (32 TOPS FP16) and Orin NX (60 TOPS FP16) can exceed 30 FPS with INT8 quantization. In contrast, Core i7/i5 remains below 20 FPS even after quantization, making it unsuitable without substantial model compression.

Additionally, when comparing various YOLOv8 and YOLOv11 variants, it is clear that model size and complexity have a direct impact on inference ability across platforms. On the discrete GPU, YOLOv8n (3.01 M params, 8.1 GFLOPs) achieves the highest FPS (129.6 FPS on RTX 3090, 109.6 FPS on RTX 1080, 100.1 FPS on RTX 3060 Laptop), closely matching YOLO11n’s performance and exceeding YOLOv8s (11.13 M params, 28.4 GFLOPs), which runs at 107.8 FPS/59.8 FPS/54.8 FPS, respectively. YOLOv8m (27.22 M params, 110.9 GFLOPs) and YOLO11m (21.58 M params, 64.9 GFLOPs) incur a larger latency penalty, dropping to 85.7 FPS/41.7 FPS/37.8 FPS (YOLOv8m) and 88.1 FPS/42.1 FPS/39.9 FPS (YOLO11m) on RTX 3090/1080/3060. This demonstrates that, on powerful GPUs, even the “m” variants can maintain well above 30 FPS, though at reduced margins.

On the Core Ultra series, the smallest variant (YOLOv8n) still runs acceptably: 47.1 FPS on Ultra 9 and 37.9 FPS on Ultra 7, indicating that the 8.1 GFLOPs cost can be handled by the integrated Xe iGPU and large L3 cache for sub-30 ms latency. By contrast, YOLOv8s’s 28.4 GFLOPs pushes both Ultra 9 and Ultra 7 to their limits—only 14.8 FPS and 11.8 FPS, respectively—falling well below near-real-time thresholds. YOLOv8m and YOLO11m similarly cannot run at all on these platforms due to memory bandwidth and cache constraints (denoted by “–”), as their 64.9 GFLOPs and 110.9 GFLOPs exceed what the heterogeneous core design can process without unacceptable stalling. YOLOv11s (21.3 GFLOPs) manages 15.9 FPS on Ultra 9 and 12.7 FPS on Ultra 7, again underperforming for most near-real-time use cases.

On conventional CPUs (Core i7/i5), only the “n” variants are feasible: YOLOv8n achieves 20.1 FPS on Core i7 and 17.8 FPS on Core i5, while YOLO11n hits 21.4 FPS and 19.6 FPS, respectively. The “s” and “m” variants of YOLOv8 and YOLOv11 all show “–” (cannot run) due to excessive computational and memory requirements. This reinforces that traditional CPUs without specialized accelerators simply cannot support these larger networks, making them unsuitable for anything beyond occasional offline inference.

The “n” variants of YOLOv8/YOLO11 can run on all tested platforms, but only on discrete GPUs and Core Ultra series do they exceed 30 FPS without quantization. The “s” variants require at least a Core Ultra 9 or higher and still fall short of real-time thresholds. The “m” variants are effectively restricted to high-end GPUs for both real-time and near-real-time applications.

Overall, our 17.7 GFLOPs model can meet or approach industrial near-real-time requirements under the hardware and optimization conditions described above. While maintaining high performance, it can also be deployed on a variety of computing devices.

#### 4.3.6. Ablation Experiments

To evaluate the effectiveness of each component under low-light conditions, we designed four ablation models (A–H) by selectively integrating or removing the IE module (LP-MSR), the DMWA module, and the MMTL module. As shown in Table 7, Model A, which incorporates all three modules, achieves the best overall performance in both detection and segmentation tasks, with detection mAP50 and mAP50-95 reaching 0.878 and 0.725 and segmentation mAP50 and mAP50-95 reaching 0.863 and 0.697, respectively—significantly outperforming other configurations.

A comparison between Models B and C reveals that removing either the IE (B) or DMWA (C) module leads to a considerable drop in detection and segmentation accuracy. This indicates that the IE module plays a crucial role in enhancing low-light imagery, while the DMWA module is essential for effective feature extraction and attention weighting under low-light conditions.

Although Model D excludes the MMTL module and thus achieves faster inference (1.5 ms), it suffers from reduced accuracy. The performance gap between Model D and the Model A confirms the importance of MMTL in boosting overall multi-task learning performance.

As shown in Table 7, in the two-way and three-way ablations (E–H), performance drops far exceed the individual ablations (B–D), indicating no antagonistic effects among modules but rather tight synergy. Specifically, removing LP-MSR and DMWA (E) or LP-MSR and MMTL (F) yields mAP50 values significantly lower than what would be expected from summing the individual ablation effects; likewise, ablating DMWA and MMTL together (G) demonstrates the loss of their collaborative gain. The three-way ablation (H) further exacerbates performance degradation, reinforcing that LP-MSR, DMWA, and MMTL collectively provide complementary benefits to detection and segmentation performance.

In summary, the collaborative integration of the IE, DMWA, and MMTL modules significantly enhances both robustness and accuracy of the model in low-light environments, thereby expanding the applicability of substation monitoring systems under challenging lighting conditions.

#### 4.3.7. Limitations and Failure Cases

**Figure 11:**
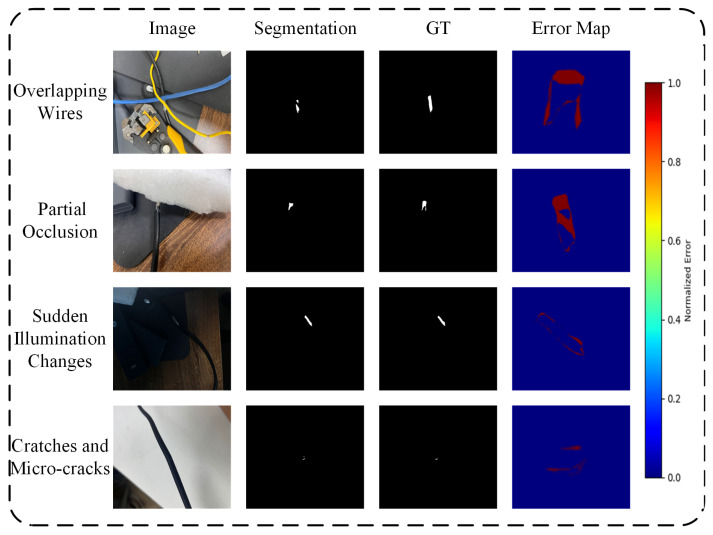
Failure cases across four extreme scenarios with segmentation results and normalized error maps. GT is binary ground-truth (GT) masks.

To investigate the limitations and failure cases, we added four extreme scenarios to the dataset—thirty images each—to more comprehensively evaluate the model under varied conditions. As illustrated in Figure 11, the first scenario is overlapping wires. When multiple wires cross and intertwine, the model struggles to distinguish each wire’s true edges from the overlapping regions, causing clear “merged” segmentations or boundary misalignments. This issue becomes especially severe when the wires have similar thickness and color against a complex background, leading the damage-detection module to mistake overlapping regions for faults and thus dramatically increasing the false-positive rate. The second scenario is partial occlusions, when a wire is partially covered by surrounding objects (e.g., foam packaging, desktop equipment, or other cables), the model cannot fully recover the features in the occluded area, resulting in “broken” or discontinuous segmentations. As a result, genuine breaks or cracks are hidden and go undetected, or intact wires are erroneously flagged as damaged. The third scenario is sudden illumination changes; for instance, under direct flashlight or strong shadow, a wire’s surface may be severely overexposed or exhibit high-contrast shadows. Thanks to our extensive lighting-augmentation strategy during training, the framework remains relatively stable in most of these conditions, preserving high-precision edges; only minor misalignments appear at extreme highlights or overexposed borders, and overall robustness is markedly better than that of conventional methods. The fourth scenario is scratches and micro-cracks. When a wire’s surface has only slight abrasions or shallow cracks, these textures closely resemble true damage contours. The model frequently misclassifies superficial scratches as damaged areas—producing false positives—while very narrow, shallow micro-cracks have low contrast and poor noise resilience, so they are often missed or incompletely segmented, leading to false negatives.

## 5. Conclusions

In this paper, we proposed a novel framework for damaged wire detection and segmentation in substations under varying lighting conditions. The framework integrates a low-light image enhancement module, an improved YOLOv11n-based detection and segmentation network with a dynamic multi-scale window attention mechanism, and a multi-stage meta-transfer learning strategy to address small-sample limitations and mitigate negative transfer. Through extensive experiments across six illumination environments ranging from 10 to 200,000 lux, it is demonstrated that our method significantly improves detection and segmentation accuracy, achieving up to 73% performance gains over baseline models while maintaining real-time inference speed.

In future work, we plan to explore the integration of multi-modal sensing to provide richer contextual information for detection and segmentation tasks. Additionally, we aim to incorporate continual learning strategies to enable long-term adaptation in dynamic and evolving environments. These enhancements will further improve the framework’s robustness, generalization capability, and practical applicability in real-world substation inspection scenarios.

## Figures and Tables

**Table 1:** Lighting settings and environment simulation.

Data Environment	Illuminance (Lux)	Images
Low Light	10–200	225
Indoor	400–600	225
Weak Cloudy Light	$1\times 10^{3}$–$8\times 10^{3}$	225
Strong Cloudy Light	$1\times 10^{4}$–$3\times 10^{4}$	225
Clear Strong Light	$5\times 10^{4}$–$8\times 10^{4}$	225
Extreme Strong Light	$1\times 10^{5}$–$2\times 10^{5}$	225

**Table 2:** Hardware and software configuration.

Category	Component
GPU	NVIDIA RTX 3090 (24 GB)
3D Camera	Mech-Mind Nova 3D Camera
Framework	PyTorch 1.8.1
System	Ubuntu 20.04 LTS
Environment	Python 3.9

**Table 3:** Comparison of detection, segmentation, efficiency, and speed across attention mechanisms on a normal-light dataset (400–600 lux). ↑ indicates that higher values are better, and ↓ indicates that lower values are better.

Method	Detection ↑		Segmentation ↑		Speed ↑	Infer ↓
	Acc. (%)	F1 (%)	Acc. (%)	F1 (%)	FPS	ms
baseline	71.30	51.04	66.40	40.25	**128.8**	**1.1**
+SE [43]	+10.33	+8.23	+9.87	+12.12	56.8	1.4
+CBAM [44]	+6.77	+4.12	+3.32	+2.58	41.7	1.8
+ECA [45]	+12.77	+10.38	+11.73	+9.89	87.9	**1.1**
+SRM [46]	+3.11	+4.22	+4.78	+5.01	67.9	**1.1**
+W-MSA [42]	+9.87	+7.87	+11.32	+9.97	39.7	1.7
+SW-MSA [42]	+13.28	+11.53	+12.97	+10.82	38.6	1.7
+DMWA	**+14.99**	**+13.54**	**+13.02**	**+11.06**	47.9	1.5

**Table 4:** Comparison of detection, segmentation, efficiency, and speed across attention mechanisms on the complex background dataset. ↑ indicates that higher values are better, and ↓ indicates that lower values are better.

Method	Detection ↑		Segmentation ↑		Speed ↑	Infer ↓
	Acc. (%)	F1 (%)	Acc. (%)	F1 (%)	FPS	ms
baseline	67.10	48.44	62.44	38.75	**128.8**	**1.1**
+SE [43]	+8.39	+7.13	+8.89	+10.98	56.8	1.4
+CBAM [44]	+6.21	+4.88	+3.66	+3.38	41.7	1.8
+ECA [45]	+10.81	+9.49	+10.67	+10.03	**87.9**	**1.1**
+SRM [46]	+4.14	+5.13	+5.07	+4.98	67.9	**1.1**
+W-MSA [42]	+9.99	+8.32	+10.16	+10.03	39.7	1.7
+SW-MSA [42]	+12.77	+10.83	+11.57	+9.93	38.6	1.7
+DMWA	**+13.76**	**+11.98**	**+12.81**	**+11.72**	**47.9**	1.5

**Table 5:** Comparison of the performances of different modules under low-light conditions (10–200 lux). ↑ indicates that higher values are better, and ↓ indicates that lower values are better.

Model	IE Method	Vision Module	Detection (↑)			Segmentation (↑)			FPS (↓)
			P	R	mAP50	P	R	mAP50	
A	Without	Yolov11n	0.417	0.455	0.433	0.285	0.337	0.312	128.8
B	Without	DMWA-MMTL (proposed)	0.763	0.791	0.786	0.761	0.767	0.774	47.9
C	Without	Yolov8n	0.408	0.443	0.426	0.277	0.328	0.306	129.6
D	Without	Yolov8s	0.613	0.563	0.596	0.497	0.513	0.499	107.8
E	Without	Yolov8m	0.747	0.771	0.754	0.752	0.721	0.744	88.1
F	Without	Yolov11s	0.662	0.689	0.667	0.575	0.523	0.538	105.4
G	Without	Yolov11m	0.764	0.775	0.767	0.747	0.797	0.775	85.7
H	LP-MSR (proposed)	DMWA-MMTL (proposed)	**0.862**	**0.881**	**0.873**	**0.861**	**0.864**	**0.869**	46.4
I	MSRCR [41]	DMWA-MMTL (proposed)	0.821	0.849	0.838	0.817	0.792	0.779	47.2
J	LLFLOW [31]	DMWA-MMTL (proposed)	0.872	0.907	0.892	0.889	0.891	0.887	21.7
K	BEM [47]	DMWA-MMTL (proposed)	0.891	0.883	0.879	0.895	0.872	0.889	17.8
L	EnlightenGAN [27]	DMWA-MMTL (proposed)	0.841	0.863	0.854	0.884	0.893	0.884	22.9
M	Py-diffusion [29]	DMWA-MMTL (proposed)	0.881	0.903	0.882	0.869	0.891	0.872	20.8
N	LP-MSR (proposed)	Yolov8n	0.598	0.623	0.609	0.489	0.518	0.499	128.7
O	LP-MSR (proposed)	Yolov8s	0.774	0.739	0.751	0.637	0.658	0.645	106.5
P	LP-MSR (proposed)	Yolov8m	0.855	0.844	0.847	0.852	0.829	0.834	86.7
Q	LP-MSR (proposed)	Yolov11n	0.603	0.635	0.621	0.481	0.533	0.502	127.4
R	LP-MSR (proposed)	Yolov11s	0.792	0.759	0.767	0.678	0.631	0.647	104.2
S	LP-MSR (proposed)	Yolov11m	0.861	0.843	0.854	0.867	0.847	0.855	**84.7**

**Table 6:** Model complexity and inference efficiency across hardware platforms. ↑ indicates that higher values are better, and ↓ indicates that lower values are better.

Hardware	Model	Params (M)	FLOPs (G)	FPS ↑	Latency ↓
RTX 3090 (24 GB)	Proposed	3.47	17.7	47.2	21.1 ms
	YOLO11n	2.59	6.9	128.8	7.7 ms
	YOLO8n	3.01	8.1	129.6	7.7 ms
	YOLO11s	9.42	21.3	105.4	9.5 ms
	YOLO8s	11.13	28.4	107.8	9.3 ms
	YOLO11m	21.58	64.9	88.1	11.4 ms
	YOLO8m	27.22	110.9	85.7	11.7 ms
RTX 1080 (8 GB)	Proposed	3.47	17.7	42.7	23.4 ms
	YOLO11n	2.59	6.9	114.3	8.7 ms
	YOLO8n	3.01	8.1	109.6	9.2 ms
	YOLO11s	9.42	21.3	60.7	16.5 ms
	YOLO8s	11.13	28.4	59.8	16.7 ms
	YOLO11m	21.58	64.9	42.1	23.8 ms
	YOLO8m	27.22	110.9	41.7	24.0 ms
RTX 3060 Laptop (6 GB)	Proposed	3.47	17.7	40.1	24.9 ms
	YOLO11n	2.59	6.9	101.9	9.8 ms
	YOLO8n	3.01	8.1	100.1	10.0 ms
	YOLO11s	9.42	21.3	57.3	17.5 ms
	YOLO8s	11.13	28.4	54.8	18.3 ms
	YOLO11m	21.58	64.9	39.9	25.1 ms
	YOLO8m	27.22	110.9	37.8	26.5 ms
Intel Core Ultra 9 (36 MB)	Proposed	3.47	17.7	26.8	37.2 ms
	YOLO11n	2.59	6.9	52.1	19.1 ms
	YOLO8n	3.01	8.1	47.1	23.2 ms
	YOLO11s	9.42	21.3	15.9	62.9 ms
	YOLO8s	11.13	28.4	14.8	67.6 ms
	YOLO11m	21.58	64.9	8.9	112.4 ms
	YOLO8m	27.22	110.9	7.1	140.8 ms
Intel Core Ultra 7 (24 MB)	Proposed	3.47	17.7	21.3	46.9 ms
	YOLO11n	2.59	6.9	39.8	25.0 ms
	YOLO8n	3.01	8.1	37.9	26.4 ms
	YOLO11s	9.42	21.3	12.7	78.7 ms
	YOLO8s	11.13	28.4	11.8	84.7 ms
	YOLO11m	21.58	64.9	6.7	149.3 ms
	YOLO8m	27.22	110.9	5.9	169.5 ms
Intel Core i7 (16 MB)	Proposed	3.47	17.7	10.9	91.7 ms
	YOLO11n	2.59	6.9	21.4	46.7 ms
	YOLO8n	3.01	8.1	20.1	49.8 ms
	YOLO11s	9.42	21.3	-	-
	YOLO8s	11.13	28.4	-	-
	YOLO11m	21.58	64.9	-	-
	YOLO8m	27.22	110.9	-	-
Intel Core i5 (12 MB)	Proposed	3.47	17.7	8.9	112.3 ms
	YOLO11n	2.59	6.9	19.6	51.0 ms
	YOLO8n	3.01	8.1	17.8	56.2 ms
	YOLO11s	9.42	21.3	-	-
	YOLO8s	11.13	28.4	-	-
	YOLO11m	21.58	64.9	-	-
	YOLO8m	27.22	110.9	-	-

**Table 7:** Detection, segmentation, and inference performance of ablation experiments under low-light conditions. ↑ indicates that higher values are better, and ↓ indicates that lower values are better.

Models	Setting				Detection (↑)				Segmentation (↑)				Inference (ms) (↓)
	Yolov	LP-MSR	DMWA	MMTL	P	R	mAP50	mAP50-95	P	R	mAP50	mAP50-95	
A	✓	✓	✓	✓	**0.867**	**0.881**	**0.878**	**0.725**	**0.864**	**0.866**	**0.863**	**0.697**	1.8
B	✓	–	✓	✓	0.759	0.787	0.783	0.644	0.758	0.761	0.779	0.583	1.6
C	✓	✓	–	✓	0.764	0.763	0.777	0.707	0.732	0.753	0.765	0.606	**1.3**
D	✓	✓	✓	–	0.813	0.832	0.804	0.741	0.828	0.833	0.821	0.656	1.5
E	✓	–	–	✓	0.637	0.659	0.644	0.534	0.597	0.588	0.572	0.501	1.3
F	✓	–	✓	–	0.646	0.671	0.651	0.539	0.611	0.603	0.609	0.526	1.3
G	✓	✓	–	–	0.603	0.635	0.621	0.472	0.481	0.533	0.502	0.409	1.2
H	✓	–	–	–	0.417	0.455	0.433	0.286	0.285	0.337	0.312	0.223	1.0

## Data Availability

The full dataset used in this study, along with the code to reproduce all experiments, will be publicly available at https://github.com/wuhan66/DMWA-MMTL (accessed on 9 June 2025) immediately upon paper acceptance.

## Glossary of Acronyms

**Acronym**	**Full Term**
YOLO	You Only Look Once
CNN	Convolutional Neural Network
DMWA	Dynamic Multi-scale Window Attention
SE	Squeeze-and-Excitation
CBAM	Convolutional Block Attention Module
ECA	Efficient Channel Attention
SRM	Style-based Recalibration Module
W-MSA	Window-based Multi-Head Self-Attention
SW-MSA	Shifted Window-based Multi-Head Self-Attention
TL	Transfer Learning
MTL	Meta-Transfer Learning
MMTL	Multi-stage Meta-Transfer Learning
LP-MSR	Light Prior-based Multi-Scale Retinex
MSR	Multi-Scale Retinex
MSRCR	Multi-Scale Retinex with Color Restoration
LLFLOW	Low-Light Flow
BEM	Bayesian Enhancement Method
EnlightenGAN	Enlighten Generative Adversarial Network
Py-diffusion	Pyramid Diffusion
RTX	NVIDIA RTX
FLOPs	Floating Point Operations
GPU	Graphics Processing Unit
IoU	Intersection over Union
mIoU	Mean Intersection over Union
P	Precision
R	Recall
mAP50	Mean Average Precision at 50% IoU
Acc	Accuracy
F1	F1-Score
